# Assessment of immunopathological responses of a novel non-chemical biocide in C57BL/6 for safe disinfection usage

**DOI:** 10.1186/s42826-024-00214-6

**Published:** 2024-08-13

**Authors:** Keun Bon Ku, Jihwan Chae, Won Hyung Park, Jeongwoo La, Seung S. Lee, Heung Kyu Lee

**Affiliations:** 1 Laboratory of Host Defenses, Department of Biological Sciences, Korea Advanced Institute of Science and Technology (KAIST), 291 Daehak-ro, Daejeon, 34141 Republic of Korea; 2 Graduate School of Medical Science and Engineering, KAIST, Daejeon, 34141 Republic of Korea; 3 Center for Infectious Disease Vaccine and Diagnosis Innovation, Korea Research Institute of Chemical Technology, Daejeon, 34114 Republic of Korea; 4 Department of Mechanical Engineering, KAIST, Daejeon, 34141 Republic of Korea

**Keywords:** In vivo, C57BL/6, Biocide, Water electrospray, Immunopathology, Lung pathology

## Abstract

**Background:**

Water electrospray technology has been developed and extensively studied for its physical properties and potential application as a non-chemical biocide against airborne pathogens. However, there are still concerns regarding the safety and potential toxicity of inhaling water electrospray (WE) particles. To address these potential hazards and offer insights into the impact of WE on humans, we analyzed the immunopathological response to WE by employing an intranasal challenge C57BL/6 mouse model. This analysis aimed to compare the effects of WE with those of sodium hypochlorite (SH), a well-known biocidal agent.

**Results:**

The study findings suggest that the WE did not trigger any pathological immune reactions in the intranasal-challenged C57BL/6 mouse model. Mice challenged with WE did not experience body weight loss, and there was no increase in inflammatory cytokine production compared to SH-treated mice. Histopathological analysis revealed that WE did not cause any damage to the lung tissue. In contrast, mice treated with SH exhibited significant lung tissue damage, characterized by the infiltration of neutrophils and eosinophils. Transcriptomic analysis of lung tissue further confirmed the absence of a pathological immune response in mice treated with WE compared to those treated with SH. Upon intranasal challenge with WE, the C57BL/6 mouse model did not show any evidence of immunopathological damage.

**Conclusions:**

The results of this study suggest that WE is a safe technology for disinfecting airborne pathogens. It demonstrated little to no effect on immune system activation and pathological outcomes in the intranasal challenge C57BL/6 mouse model. These findings not only support the potential use of WE as an effective and safe method for air disinfection but also highlight the value of the intranasal challenge of the C57BL/6 mouse model in providing significant immunopathological insights for assessing the inhalation of novel materials for potential use.

**Supplementary Information:**

The online version contains supplementary material available at 10.1186/s42826-024-00214-6.

## Background

Over the centuries, the emergence and spread of novel infectious diseases have led to several pandemic outbreaks with devastating consequences [1, 2]. Pathogens such as plaque, influenza A, Middle East respiratory syndrome coronavirus (MERS-CoV), severe acute respiratory syndrome coronavirus (SARS-CoV), and *Mycobacterium tuberculosis* are the major pandemic pathogens known to spread through the air. They can infect and be transmitted through the human respiratory tract [3–5].

To combat these deadly pathogenic microbes, preventative and therapeutic strategies are essential. These strategies include utilizing diagnostic tools, antimicrobial drugs, and vaccine injections [6, 7]. However, since these strategies require development time due to their pathogen-specific nature, other methods such as the use of disinfectants for personal and public hygiene are essential for controlling and slowing down the spread of pathogens in the early stages of the pandemic [8, 9]. The importance of this simple yet effective preventive action has grown, especially after the COVID-19 outbreak, when individuals may face difficulties accessing drugs and vaccines due to disruptions in the healthcare system [10, 11].

Antimicrobial biocides are widely used as disinfectants for personal and public hygiene. Most of them are chemical agents such as alkylating agents (e.g., formaldehyde), oxidizing agents (e.g., sodium hypochlorite, hydrogen peroxide, and povidone-iodine), cationic agents (e.g., quaternary ammonium compounds, biguanides, diamines, and amine oxides), phenolics (e.g., triclosan), and alcohols (e.g., ethyl alcohol and isopropyl alcohol) [10, 12]. The majority of antimicrobial biocides primarily function by disrupting the integrity of the cytoplasmic membrane of pathogens [10] and are predominantly used for disinfecting surfaces, water, and equipment. They are not typically used for air disinfection because chemical biocides can be harmful to humans, especially to the respiratory tract [13, 14]. However, since most pandemic pathogens are airborne diseases, they effectively transmit through the air in droplet or aerosol forms. Thus, disinfection of the air containing pathogenic particles is an important strategy for controlling the transmission of pathogens.

Given these circumstances, the development of air disinfectants is imperative, and water electrospray (WE) emerges as a promising option for disinfecting airborne pathogens. Water electrospray technology, first introduced by Pantano et al. [15], produces charged aerosols with antimicrobial activity by applying a high voltage to water through a micro-sized nozzle [16, 17]. As a chemical-free agent, it has been proven effective in eliminating pathogens [18, 19]. A key factor in its biocidal efficacy is the production of OH radicals, a critical subset of reactive oxygen species (ROS) known for their high reactivity and ability to kill microbes. These radicals effectively target a broad spectrum of pathogens in the air. Thus, WE, a non-chemical biocide, emerges as an effective disinfectant for airborne pathogens. Its significance is increasing, making it an attractive strategy for preventing new outbreaks of various pathogens.

However, the safety and toxicity of WE must be validated before human application. Although one study has investigated the toxicity of WE [19], it only considered short durations (several hours) of the mouse challenge condition and a limited set of hazard parameters, focusing solely on bronchoalveolar lavage (BAL) fluid. Our study addressed these limitations by analyzing the effects of WE over an extended period, with repeated airway exposures over a week, using a C57BL/6 mouse intranasal challenge model to replicate both acute and sub-chronic (or sub-acute) exposures [20, 21]. From the results, we found that WE, a non-chemical biocide, demonstrated no adverse impact on the immune system in this intranasal challenge C57BL/6 mouse model. These findings suggest that WE could be a safe option for disinfecting airborne pathogens, supporting its potential application for human use.

## Methods

### Mice

Male C57BL/6 mice aged 7–9 weeks were used for the experiments. All animals were housed in a climate-controlled, pathogen-free facility at the Korea Advanced Institute of Science and Technology (KAIST) with ad libitum access to food and water under a 12-h light/dark cycle. All animal procedures were conducted following protocols approved by the KAIST Institutional Animal Care and Use Committee (approval code: KA2022-065-v1).

### Generation of water electrospray (WE)

To produce the WE, we utilized a custom-fabricated polymer nozzle specifically designed for electrospray applications. The nozzle featured a single pyramid-shaped orifice, 60 μm in diameter, that was integrated with an in-plane extractor. The in-plane extractor, implemented as a ring-shaped electrode around the nozzle, was grounded to concentrate the electric field and maximize the electrospray effect. The electrospray was generated in a simple jet mode, propelling triple-distilled water at a flow rate of 50 ml/h using a syringe pump. A negative voltage of − 1.5 kV was applied to the water, resulting in a stable and uniform electrospray of microdroplets. The grounded aluminum foil collector was positioned 5 cm away from the nozzle to effectively collect the aerosolized droplets. This configuration was selected to optimize both the production and collection of a steady stream of fine droplets. For detailed specifications and operational parameters of the custom polymer nozzle and in-plane extractor system, readers are referred to our previous work [16].

### Bacterial strains and growth conditions

The bacterial strains utilized in this study were obtained from the Korean Collection for Type Cultures (KCTC). *Escherichia coli* (KCTC 1682) cultured in Luria Bertani broth (LB-05, LPS Solution, Korea) at 37 °C with 300 rpm shaking was employed for bactericidal effect testing. Bacterial colony-forming units (CFU) were quantified using the spread plate method. *Escherichia coli* cells were spread on LB agar and incubated at 37 °C for 24 h. Subsequently, all the titers were quantified by counting the number of colonies.

### Bactericidal effect test

After reaching a growth density of 1.4 × 10^9^ CFU/ml (equivalent to 0.26 OD600), bacterial cells were harvested by centrifugation at 15,000 rpm for 5 min and adjusted to a concentration of 1 × 10^3^ CFU/ml in Dulbecco’s phosphate-buffered saline (DPBS, 21-031-CV, Corning Inc., Corning, NY, USA). These bacterial samples were then mixed with a 10 × volume of freshly collected WE, sterile distilled water, 3% sodium hypochlorite solution (SH, Sigma-Aldrich, St. Louis, MO, USA), or LB broth. After 1 or 2 h of room temperature incubation, the cells were serially diluted in 1 × DPBS and then spotted on LB agar to count the surviving bacteria. After an overnight incubation at 37 °C, the colonies were counted to determine the bactericidal reduction rate. Triplicate plates were used in the titration of the mixture.

### In vivo challenge

To assess the immune response and histopathological changes following WE treatment, a mouse challenge model was employed. Twenty microliters of freshly collected WE, sterile DW, or 0.01% SH were administered intranasally. In the acute challenging condition, the mice were treated three consecutive times. In the sub-chronic challenging condition, the mice were treated five times with three days between each treatment within 2 weeks [22, 23]. Throughout the experiment, the mice were monitored for changes in body weight and clinical symptoms. Seven days after the final treatment for each challenging condition, all the mice were euthanized to collect lung tissue.

### Enzyme-linked immunosorbent assay (ELISA)

After challenging the mice with a bactericidal agent WE, SH, or DW, they were euthanized, and their bronchoalveolar lavage fluid was collected at a specified time point. For the BAL fluid collection, the thorax was opened, and a 20-gauge intravenous catheter (3S-CAT 20G, Dukwoo Medical, Korea) was inserted into the trachea through the cricothyroid membrane. The trachea was then flushed with 800 μl of cold PBS, and the collected BAL fluids were stored at − 80 °C. The levels of IL-1β (R&D, DY401), IL-6 (R&D, DY406), and IL-12p40 (R&D, DY499) in the BAL fluids were measured using ELISA kits following the manufacturer’s protocols. TNF-α in the BAL fluid was detected using a sandwich ELISA assay with purified anti-mouse TNF-α detection antibodies (Biolegend, 510805) and biotinylated anti-mouse TNF-α antibodies (Biolegend, 506312).

### Histopathological analysis

The mice were euthanized at the designated time points, and their lung samples were fixed in 4% paraformaldehyde. Subsequently, the lung tissues were embedded in paraffin blocks for histological processing. Thin sections (approximately 5 μm thick) were prepared from the paraffin-embedded blocks and mounted on glass slides. These sections were then subjected to hematoxylin and eosin (H&E) staining to facilitate the examination of histopathological alterations. Three animals per experimental group, and three images from each individual, were included in the analysis. The lung tissue sections were examined using a digital slide scanner (3D Histech, Budapest, Hungary), and the severity of the tissue damage was assessed. A grading system ranging from 0 to 4 was employed to quantify the extent of damage, representing no observed histopathological changes (0) or mild (1), moderate (2), severe (3), or diffuse (4) levels of observed histopathological changes.

### Lung immune cell isolation, staining, and flow cytometry

To isolate single lung immune cells, we used a Lung Dissociation Kit (Miltenyi Biotec, 130-095-927) and GentleMACS Octo Dissociator (Miltenyi Biotec) following the manufacturer’s instructions. The immune cell suspensions were first passed through a 70-μm cell strainer, then enriched in mononuclear cells using a Percoll density gradient (GE Healthcare Life Sciences, 17-0891-01), and treated with an ammonium–chloride–potassium (ACK) lysis buffer at room temperature to eliminate red blood cells. Staining reactions were performed at 4 °C in FACS (Fluorescence-activated cell sorting) buffer (PBS containing 1% BSA [Millipore, 82-100-6], 1% penicillin/streptomycin [GenDEPOT, GED-CA005-010]) with 1% v/v of anti-CD16/32 antibody (2.4G2) to block Fc receptors and reduce non-specific binding. The following antibodies were used for flow cytometry: CD45.2-PE (eBioscience; clone 104), CD19-PerCP/Cy5.5 (BioLegend; clone ID3), CD44-APC (BioLegend; clone IM7), CD8α-APC/Cy7 (BioLegend; clone 53-6.7), NK1.1-PE/Cy7 (BD; clone PK136), CD3-Alexa Fluor 700 (BioLegend; clone 17A2), CD4-BV421 (BD; clone GK1.5), MHCII-PerCP/Cy5.5 (BioLegend; clone M5/114.15.2), Ly6G-APC (BioLegend; clone 1A8), Ly6C-APC/Cy7 (BioLegend; clone HK1.4), CD11c-PE/Cy7 (BioLegend; clone N418), CD11b-Alexa Fluor 700 (BioLegend; clone M1/70), and Siglec-F-BV421 (BD; clone E50-2440). Zombie Aqua™ Fixable Viability Dye (BioLegend, 423101) was used to distinguish between dead and live cells. A BD FACS LSR Fortessa 3 flow cytometer was used to acquire over 100,000 events, and the data were analyzed using FlowJo.

### Bulk RNA sequencing

After the mice were euthanized using CO_2_, the pulmonary vasculature was perfused with cold PBS through the right ventricle. The thoracic cavity was dissected, and the lungs were removed and placed in a 50 ml test tube containing 2.5 ml of RNAlater (ThermoFisher, AM7020, Waltham, MA, USA). After 24 h of incubation, an ethanol wash was performed, and the total RNA was isolated from the lungs using RNeasy mini kits (QIAGEN, 74106, GmBH, Germany). The quality analysis and sequence analysis were performed by Macrogen Inc. (Seoul, Korea). In brief, the quality of each RNA sample was assessed using the Agilent 2100 Bioanalyzer (Agilent) with a DNA 1000 chip. For RNA sequencing, 1 μg of the total RNA was analyzed using the TruSeq RNA library kit and reverse transcribed into cDNA libraries. The procedure involved the extraction of polyA-selected RNA, followed by RNA fragmentation, reverse transcription with random hexamer priming, and sequencing with 100-nt paired-end reads using an Illumina HiSeq2000 (Illumina, San Diego, CA, USA).

### Analysis of bulk RNA sequencing data

First, we pre-filtered genes with undetected levels. Next, data normalization was performed using DESeq2. Differentially expressed genes (DEGs) were identified using the DESeq2 (version 1.34.0) R package to identify statistically significant and biologically meaningful expression differences. In this analysis, genes with an adjusted *p-*value less than 0.005 and |log2FC| greater than 0.5 were selected as DEGs. The R packages factoextra (version 1.0.7), Enhanced Volcano (version 1.12.0), and fgsea (version 1.29.1) were used for data analysis and visualization., The gene set enrichment analysis was conducted using and the Hallmark, BioCarta, KEGG, Reactome, GO: BP, and GO: MF from the Mouse Molecular Signatures Database(Mouse MSigDB).

### Statistics

The statistical analysis was conducted using GraphPad Prism 9 software (GraphPad Software, La Jolla, CA, USA). The data are presented as mean ± SEM. Sample data with a normal distribution were analyzed for statistical significance (*: *p* < 0.05, **: *p* < 0.01, ***: *p* < 0.001, ****: *p* < 0.0001, ns: not significant) using one-way or two-way ANOVA tests for multiple-group comparisons and Student’s t-test for two-group comparisons.

## Results

### Bactericidal effect of water electrospray (WE)

**Fig. 1:**
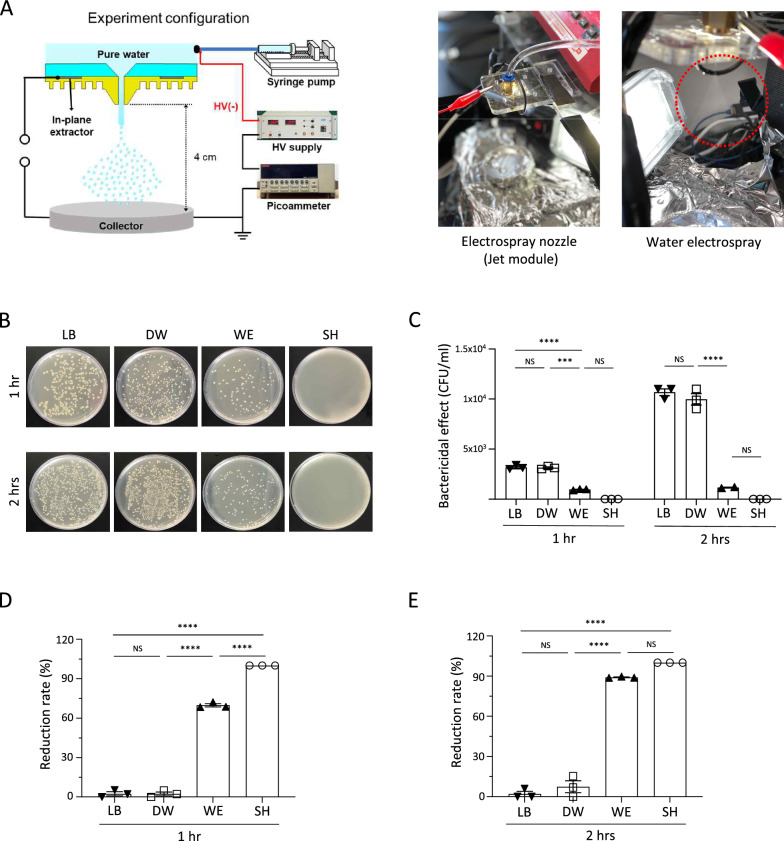
Bactericidal effect of water electrospray against *E.coli*. The bactericidal effect of water electrospray (WE) produced by a prototype laboratory-designed electrospray module was assessed by measuring the reduction rate of *E. coli*. **A** Experimental configuration of the WE–generating devices, consisting of a high voltage (HV) supply, pico ammeter, syringe pump, jet module, and collecting tray. The red circle indicates the ejecting WE from the jet module. **B**, **C** Colony counts analyzed the bactericidal effect. The process involved diluting the mixtures by 10^1^- or 10^2^-fold, followed by incubation for 1 or 2 h. Each tenfold diluted sample was plated in triplicate, and after 24 h of incubation, the colonies were counted. **B** Representative images of agar plates used for colony counting. **C** Bacterial titer (CFU/ml) of each mixture. **D**, **E** Colony reduction rate based on LB broth–conditioned *E. coli*. Results of incubation at room temperature for **D** 1 h and **E** 2 h. The bar plots show the mean ± SEM; the dots represent individual biological replicates. *p* values are from one-way or two-way ANOVA. *p < 0.05, **p < 0.01, ***p < 0.001

To assess the antibacterial efficacy of WE, the gram-negative bacterial strain *E. coli* was chosen as the test pathogen [24]. The antibacterial effectiveness of WE was evaluated in comparison to the activity of a commonly used bactericidal agent, 3% SH [25, 26]. The WE used in the study was freshly produced using a laboratory-designed electrospray module, which includes a water-ejecting syringe pump, an electric generator, and an electrospray nozzle. The WE produced exhibited a visibly sprayed appearance in the form of fine droplets (Fig. 1A) and tested the bactericidal effect of WE followed by the method described in the methods section. In the results the LB broth control group, the tested bacteria showed growth with room temperature incubation, and the use of DW did not impact bacterial growth or viability (Fig. 1B). Treatment with SH sterilized the bacteria after 1 and 2 h of incubation, while WE exhibited bactericidal activity after 1 h and further reduced colony numbers after 2 h (Fig. 1B). The WE sterilized and inhibited the growth of *E. coli*, as indicated by the titer (CFU/ml) of the mixture (Fig. 1C). The bactericidal effect of WE was also evaluated by calculating the reduction rate [27, 28]. After a 1-h incubation of *E. coli* with WE, there was a 69.8% reduction rate. Following a 2-h incubation, the reduction rate increased to 89% compared to the LB broth control (Fig. 1D, E). Although the bactericidal effect of the WE was less potent than that of SH at 1 h, there were no significant differences in their reduction rates after a 2-h incubation (Fig. 1D, E). Notably, the extended incubation period with WE resulted in an enhanced bactericidal effect. Taken together, the intrinsic electrical properties of freshly produced WE maintained its antibacterial qualities for up to 2 h at room temperature, demonstrating a significant bactericidal effect and indicating its suitability as a disinfectant.

### Absence of clinical symptoms and proinflammatory cytokines after challenge with WE

**Fig. 2:**
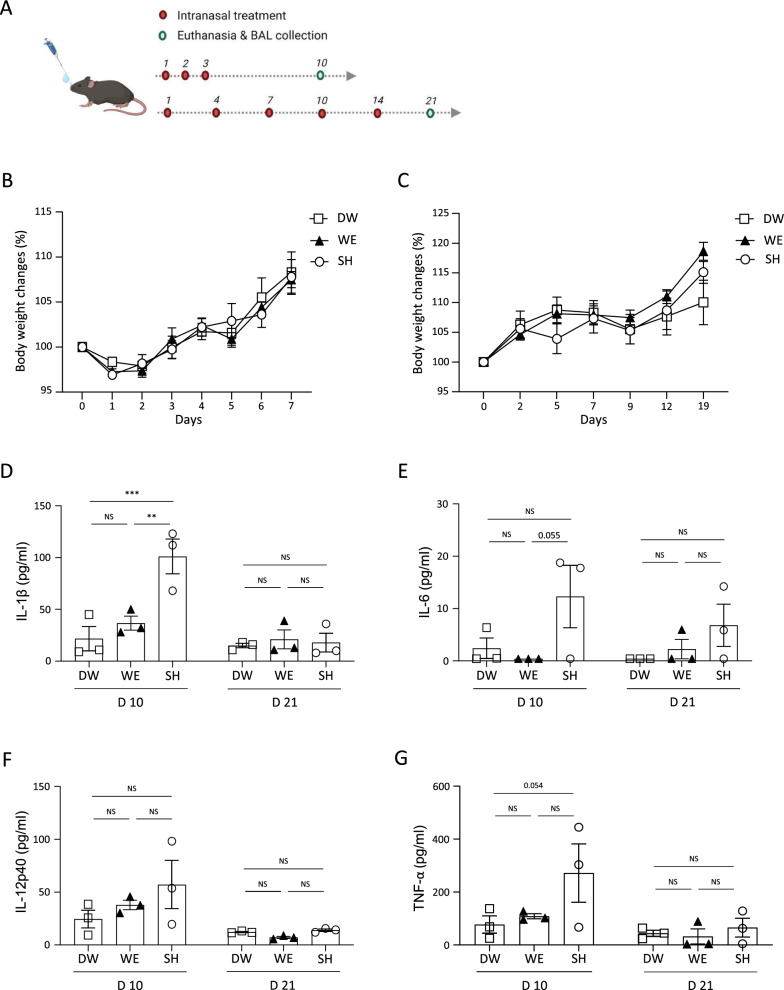
Analysis of weight and inflammatory cytokines changes of WE–treated mice. Body weight changes and inflammatory cytokines from bronchoalveolar lavage (BAL) fluid were analyzed in mice treated with distilled water (DW, n = 3), water electrospray (WE, n = 3), and sodium hypochlorite (SH, n = 3). **A** Experimental scheme of the treatment and BAL collection. **B**, **C** After the intranasal treatment of the agents, body weight changes were monitored for the groups challenged **B** acute and **C** sub-chronic challenging condition. **D**–**G** Levels of mouse cytokines in BAL fluids collected at both 10 days and 21 days from treated mouse groups: **D** IL-1β, **E** IL-6, **F** IL-12p40, and **G** TNF-α. The bar plots show the mean ± SEM; the dots represent individual mice. *p* values are from one-way (**D**–**G**) or two-way ANOVA (**B**, **C**). *p < 0.05, **p < 0.01, ***p < 0.001

In addition to evaluating the antimicrobial properties of WE, we aimed to investigate its potential in vivo toxicity and immunological response by employing a C57BL/6 mouse model and administering it intranasally. The experimental design included two challenge conditions (Fig. 2A): (1) acute challenging condition, involving three consecutive administrations with sample collection on the 10th day, and (2) sub-chronic challenging condition, consisting of five administrations with a 3-day interval and sample collection on the 21st day. Freshly obtained WE was used for the treatment group, while DW and SH were used for the control groups. SH is widely used as an antimicrobial agent [26], but it has severe harmful effects on the human body, particularly when inhaled [29, 30]. Consistent with toxic-related references, we also observed that 0.1% SH resulted in 100% fatality with severe lung damage (data not shown). Therefore, we chose to use a sublethal dose of 0.01% of SH as the control agent. Following the administration of the test substances—WE, DW, and SH—no significant changes in body weight were observed until the collection of BAL fluid (Fig. 2B, C). The presence of proinflammatory cytokines in the airway tract was examined by analyzing the BAL fluid. Specific proinflammatory cytokines that could be induced under immunopathogenic conditions of acute lung injury, such as exposure to chemical toxins, including IL-1β, IL-6, IL-12p40, and TNF-α, were quantified [31–33]. Under sub-chronic challenging conditions, all the cytokines measured from the BAL were comparable among all the groups: DW, WE, and SH (Fig. 2D–G). On day 10 after inducing an acute challenging condition, the secretion of inflammatory cytokines in the BAL samples from the WE-treated group was comparable to that of the DW-treated group. However, in the case of SH, a significant increase in IL-1β secretion was observed (Fig. 2D). IL-6 expression was not significantly different between the DW and SH groups; however, IL-6 expression was significantly higher in the SH group compared to the WE group (Fig. 2E). Moreover, the mice treated with WE showed low levels of IL-12p40 on day 10, similar to those in the DW group. By contrast, mice treated with SH exhibited a general increase in measured IL-12p40 values, although it did not reach statistical significance (Fig. 2F). TNF-α increased by day 10 in the SH-treated mice with a marginal *p*-value, whereas the WE, as expected, did not result in an increase in TNF-α levels in the BAL fluids. Although the treatment with sublethal doses of SH did not result in clinical symptoms such as changes in body weight, the respiratory tract underwent inflammatory changes characterized by the secretion of proinflammatory cytokines IL-1β, IL-6, and TNF-α. In contrast, the mice treated with WE showed an absence of proinflammatory cytokines in their BAL samples. Taken together, these data indicate that inhaled WE did not induce any clinical symptoms or proinflammatory cytokine production in the lungs.

### Histopathological analysis of lung tissue revealed minimal tissue damage in response to WE challenge

**Fig. 3:**
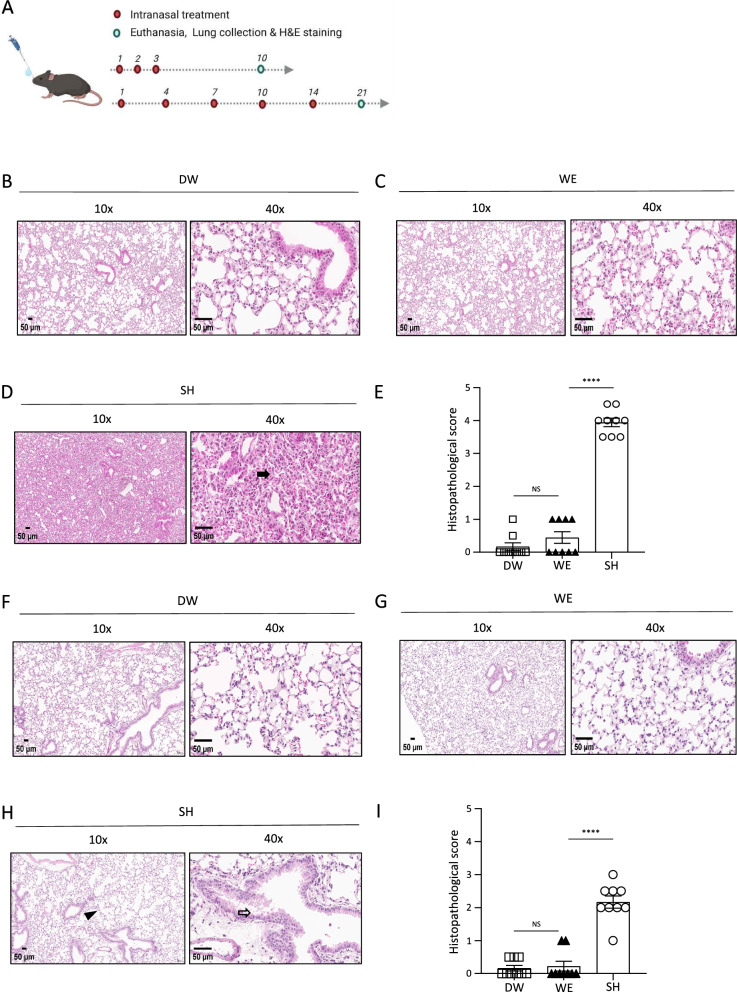
Histopathological analysis of WE–treated mice. Histopathological analysis was conducted using H&E staining. Distilled water (DW, n = 3), water electrospray (WE, n = 3), and sodium hypochlorite (SH, n = 3) were administered for the treatments. **A** The experimental schedule of the treatments and lung H&E staining was established. **B**–**E** Representative images of H&E-stained lungs were captured 10 days after treatment with **B** DW, **C** WE, or **D** SH (closed arrow highlights immune cell infiltration). For the **E** pathological score, three images from three individual mice were analyzed. **F**–**I** Histopathological images on day 21 were analyzed in the same manner. Representative images following treatment with **F** DW, **G** WE, and **H** SH are depicted (arrowhead indicates emphysema-like alveoli, and closed arrows highlight goblet cell hyperplasia). **I** Pathological scores were analyzed. The bar plots show the mean ± SEM; the dots represent individual images from a treated group. *p* values are from one-way ANOVA. * *p* < 0.05, ** *p* < 0.01, *** *p* < 0.001

To assess potential respiratory tissue damage, we conducted a histopathological analysis of the lung tissue collected after administering the agents under two different intranasal challenge conditions (Fig. 3A). The H&E-stained images from three different sections of three mice for each type of agent were assessed (Supplementary Fig. 1). Under the acute challenging condition, the group treated with DW showed intact alveolar structures with well-defined, segmented air spaces. The bronchioles exhibited proper layers of basal and goblet cells, indicating normal physiological structures (Fig. 3B). Similar to the DW group, the group treated with WE exhibited intact alveolar structure with minimal immune cell infiltration (Fig. 3C). By contrast, the group treated with SH showed severe lung tissue damage, characterized by congested alveoli with inflammatory immune cells and red blood cells, and the bronchioles underwent narrowing due to immune cell infiltration in the surrounding tissue (Fig. 3D). The selected images (n = 9) from each group were scored based on the histopathological grade (Supplementary Table 1) [32]. The SH–treated mice exhibited a high histopathological score of approximately 4, whereas all the mice treated with DW or WE achieved scores of 0 or close to 0, respectively (Fig. 3E). Notably, the WE–treated group showed a low histopathological score of 0.4, suggesting a minimal harmful effect on lung tissue. In the sub-chronic challenging condition, the group treated with WE exhibited preserved histological structures, similar to the group treated with DW (Fig. 3F, G). In contrast, the mice treated with SH exhibited emphysema-like alveolar structures, along with hyperplasia of goblet cells and mucus accumulation in the bronchioles (Fig. 3H). The lung images were also analyzed for histopathological scores, revealing a higher score of 2.1 points in the SH-treated mice, while the WE group showed a lower score of 0.2 (Fig. 3I). The results of the histopathological analysis indicate that intranasal treatment with WE did not damage the lung tissues, while SH severely damaged the lung tissue under the same treatment conditions.

### Lung resident and infiltrating myeloid immune cells were changed in pathological lung tissues, and WE-challenged mice had nonpathological immune cell composition

**Fig. 4:**
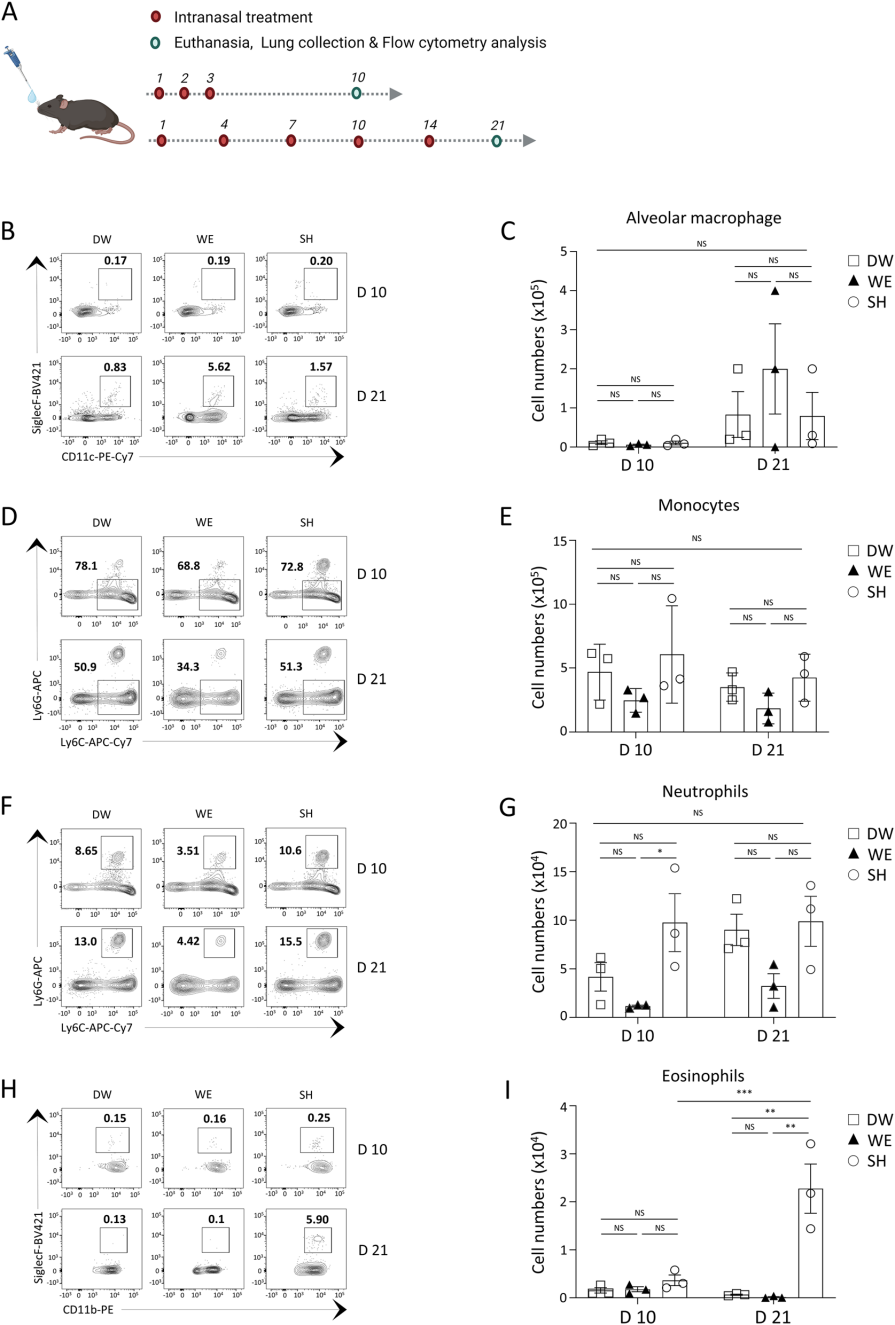
Lung tissue–infiltrating myeloid immune cell analysis of WE–treated mice. The lung resident and infiltrating immune cells of mice treated with distilled water (DW, n = 3), water electrospray (WE, n = 3), and sodium hypochlorite (SH, n = 3) were analyzed using flow cytometry. **A** Experimental schedule and FACS analysis of lung resident and infiltrating immune cells. **B**, **C** Alveolar macrophages were quantified in lung tissues harvested at 10 days and 21 days. **B** Representative FACS plots of SiglecF^+^ CD11c^+^ alveolar macrophages and **C** total cell count. **D**, **E** Lung-infiltrating monocytes were assessed by Ly6C^+^ and Ly6G^−^. **D** Representative FACS plots and **E** total cell count of monocytes. **F**, **G** Lung-infiltrating Ly6C^+^ Ly6G^+^ neutrophils were quantified. **F** Representative FACS plots and **G** total cell count of neutrophils. **H**, **I** Lung-infiltrating SiglecF^+^ CD11b^+^ eosinophils were measured. **H** Representative FACS plots and **I** total cell count of lung tissue–infiltrating eosinophils. The bar plots show the mean ± SEM; the dots represent individual mice. *p* values are from two-way ANOVA. *p < 0.05, **p < 0.01, ***p < 0.001

In human cases, acute lung injury (ALI) is characterized by acute lung inflammation, which leads to reduced oxygen exchange in the alveoli. ALI represents an early stage of the condition, whereas acute respiratory distress syndrome (ARDS) is a more severe and advanced form of ALI [34, 35]. The dose and concentration selected for the intranasal challenge of C57BL/6 mice were determined based on body weight changes, production of inflammatory cytokines, and histopathological findings. These parameters were carefully chosen to induce ALI without progressing to severe ARDS, thereby preventing mortality in the mice. ALI can be induced by various factors, including physical or chemical irritation, as well as infections [34, 35]. The immune response in the respiratory tract varies depending on the type of irritation. To analyze the immunopathological response based on the biocides, immune cell analysis was conducted on lung tissues treated with the agents under two types of challenging conditions (Fig. 4A). Tissue-resident immune cells, specifically alveolar macrophages, play a key role in maintaining immune homeostasis in the respiratory tract [36, 37]. Under the acute challenge condition on day 10, there was no noticeable difference in the number of alveolar macrophages among all groups. By day 21, under the sub-chronic challenging condition, there was a general increase in the average total number of alveolar macrophages in all groups compared to day 10 of the acute challenging condition. Despite this increase, the quantity of alveolar macrophages remained similar across all groups (Fig. 4B, C). Lung-infiltrating blood-derived monocytes also showed similar total cell numbers in the lung tissue at 10 days and 21 days across all groups (Fig. 4D, E). Additionally, neutrophil and eosinophil recruitment in the lung tissue was analyzed, as these immune cells play pivotal roles in the lung pathology of chemically induced or allergic asthma [38–40]. The lung tissue samples collected 10 days after SH treatment exhibited an increased total number of neutrophils compared to the WE–treated mice. By day 21, lung neutrophils were recruited in all groups, and the total cell numbers were comparable among the groups (Fig. 4F, G). The group treated with SH also showed an increase in eosinophils at day 21; however, WE–treated mice had basal levels of eosinophil infiltration (Fig. 4H, I). In contrast to the significant recruitment of inflammatory immune cells such as neutrophils and eosinophils observed in response to SH treatment, the WE challenge showed minimal alterations in immune cell infiltration. Specifically, the levels of pathological myeloid immune cell recruitment in mice subjected to WE were found to closely resemble those in the DW challenge group.

There were no significant differences found in the lymphoid immune cells among all the groups on day 10 (Supplementary Fig. 2A, B, E, F, I, J). However, on day 21 (Supplementary Fig. 2C, D, G, H), the groups of mice treated with WE showed a decrease in T-cell infiltration compared to the other groups (Supplementary Fig. 2K, L). Although the reduction in the number of T cells at day 21 did not seem to affect the lung tissue pathology according to the histological results, this decrease still needs to be taken into consideration.

### WE-challenged mice exhibited minimal lung tissue transcriptome alterations

**Fig. 5:**
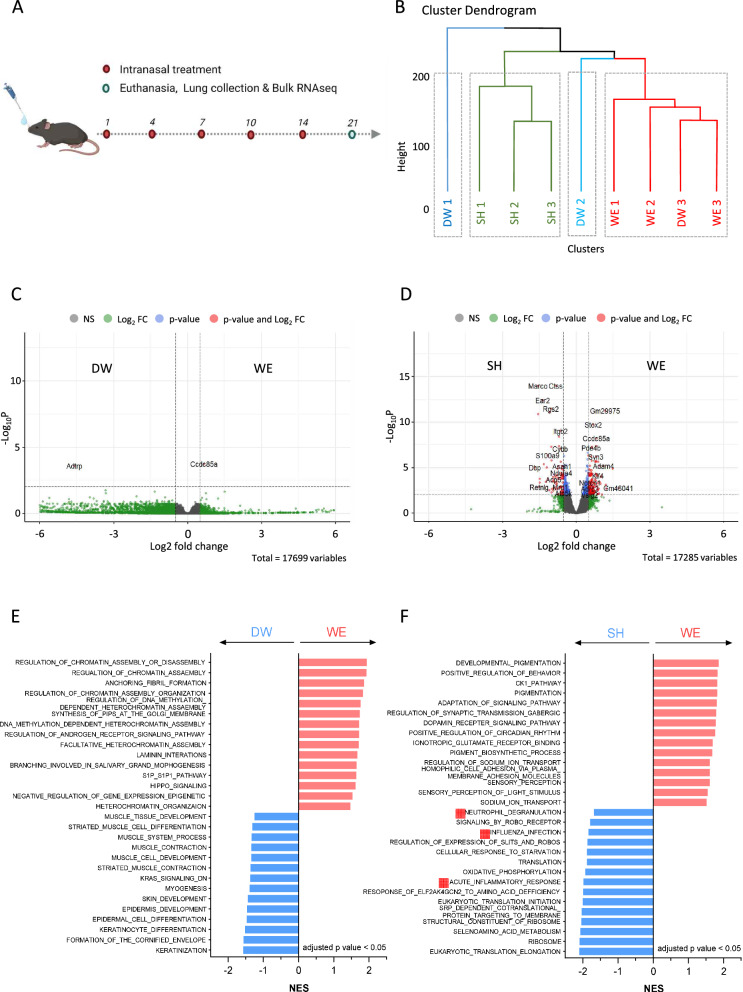
Bulk RNA sequencing analysis of lung tissue from WE–treated mice. To analyze transcriptome-level changes in mice challenged with water electrospray (WE, n = 3) compared with those treated with distilled water (DW, n = 3) or sodium hypochlorite (SH, n = 3) at 21 days, total RNA was extracted from their lungs. Subsequently, bulk RNA sequencing was conducted to investigate and analyze the transcriptome-level changes. **A** An experimental schedule was established for the treatment and transcriptome analysis of lung tissues. **B** Dendrogram of hierarchical clustering of DW–, WE–, and SH–treated mice. **C**, **D** Volcano plots were generated to visually depict the differentially expressed genes between the **C** WE versus DW and **D** WE versus SH groups (cut-off value: − log_10_P < 0.001 and fold change > 0.5). **E**, **F** Gene set enrichment analysis was performed to identify enriched gene sets between the **E** WE versus DW and **F** WE versus SH groups. A list of enriched gene sets with an adjusted *p*-value < 0.05 was generated

In a sub-chronic challenging condition, whole lung tissue was collected for RNA purification. Transcriptome changes at the tissue level were assessed in mice challenged with WE-treated mice, comparing them to groups treated with DW- or SH-treated mice (Fig. 5A). In the dendrogram analysis of hierarchical clustering, it was noted that the WE group showed low similarity with the SH group, whereas specific DW groups displayed high similarity with the WE group (Fig. 5B). In the differential expression gene (DEG) analysis comparing the WE and DW groups, only two genes, namely androgen-dependent TFPI-regulating protein (*Adtrp*) and coiled-coil domain-containing 85A (*Ccdc85a*), were identified (Fig. 5C). A considerable number of genes were identified in the DEG of the WE and SH groups, with some of these genes being associated with lung pathology (Fig. 5D). Cathepsin S (*Ctss*) is a gene associated with lung inflammation in ARDS [41], and the class A scavenger receptor, macrophage receptor with collagenous structure (MACRO), is related to genes associated with lung damage [42, 43]. Eosinophil cationic protein 2 (*Ear2*) [38] and calprotectin (*S100a9*) [44, 45] levels were elevated in the SH groups compared to the WE groups, corresponding to an increased infiltration of eosinophils and neutrophils (Fig. 5D).

A gene set enrichment analysis was conducted to explore functional pathways using multiple databases, including BioCarta, GO: BP, GO: MF, Hallmark, KEGG, and Reactome. Enrichment analysis of the groups treated with DW and WE did not reveal any gene sets related to inflammatory immune responses. In the mice treated with DW, gene sets related to tissue biogenesis such as “KERATINIZATION”, “SKIN_DEVELOPMENT”, and “MUSCLE_TISSUE_DEVELOPMENT” were enriched. Additionally, the mice treated with WE showed enrichment in the “ANCHORING_FIBRIL_FORMATION” gene set. Anchoring fibril formation is the biological process of connecting the dermis and epidermis through the accumulation of collagenase VII. Anchoring fibril formation is possible in the lungs with fibrotic tissue formation [46, 47]. Thus, it suggests that although the protein levels were not transduced, the transcriptome level may change due to the sub-chronic challenging condition of WE (Fig. 5E, Supplementary Fig. 3). The SH–treated mice showed enrichment in gene sets associated with inflammation, such as “ACUTE_INFLAMMATORY_RESPONSE”, “INFLUENZA_INFECTION”, and “NEUTROPHIL_DEGRANULATION”. In contrast, the mice treated with WE did not show enrichment in immune response-related gene sets. Instead, they exhibited enhanced pigmentation-related gene sets such as “DEVELOPMENTAL_PIGMENTATION”, “PIGMENTATION”, and “PIGMENT_BIOSYNTHETIC_PROCESS” (Fig. 5F). In summary, the gene set enrichment analysis revealed that DW and WE treatments did not induce inflammatory immune responses. DW-treated mice showed enrichment in tissue biogenesis gene sets, while mice with WE showed enrichment in anchoring fibril formation gene sets. In contrast, SH-treated mice exhibited gene sets associated with inflammation, which contrasted with the pigmentation-related gene sets enriched in WE-treated mice.

## Discussion

Given the increasing demand for novel biocides to control airborne pathogens, WE has emerged as a promising solution. However, a thorough investigation into its toxicity characteristics is essential, especially when considering its potential use for disinfecting air containing aerosolized pathogens that could inadvertently be inhaled by humans. To evaluate the toxicity and safety of WE following human inhalation at the preclinical stage, we utilized a C57BL/6 mouse model for repeated intranasal challenges with WE.

Mice challenged with WE or SH did not exhibit any body weight loss. However, the mice treated with SH showed increased expression of IL-1β, IL-6, and TNF-α, as well as severe lung inflammatory histopathology on day 10. In contrast, the mice treated with WE did not show any immunopathological findings in the BAL fluid or lung images. Additionally, analysis of immune cells in the lung revealed that in the SH–treated mice, neutrophils and eosinophils were significantly increased in lung tissue on days 10 and 21, respectively. These pathological features are strongly associated with pulmonary inflammation and resemble lung damage observed in chemically induced asthma [48, 49]. By contrast, the group of mice that underwent the same treatment schedule with WE did not exhibit inflammatory immune cell infiltration. However, a decrease in T cells was detected in the lung tissue at day 21 compared with DW. The reduced number of T cells in WE-treated mice requires further investigation to confirm its effect on specific T cells.

The transcriptomic analysis revealed differential expression of gene sets between the groups. As expected, mice treated with SH exhibited an increase in inflammation-related gene sets, while the group treated with WE did not show enrichment of inflammation-related gene sets. Furthermore, the WE–treated mice showed enrichment of pigmentation-related gene sets compared to the SH–treated mice. Excessive pigmentation of the lung tissue is a known inducer of phagocytosis by alveolar macrophages during the process of clearing damaged tissue [50]. However, since mice treated with WE did not show an increase in phagocytic immune cells or tissue damage, the upregulation of pigmentation-related gene sets is unlikely to be a result of lung tissue damage. The WE–treated mice also exhibited enrichment in the gene set related to fibril formation, which could potentially contribute to the development of fibrosis-related lung pathogenesis [46]. The reasons and consequences of the enrichment of pigmentation-related and fibril formation genes in the lung warrant further investigation to ensure the safety of WE.

In this study, we focused on evaluating the immunopathological effects of WE on the lungs through ELISA, FACS, and transcriptome analysis. However, to ensure the safe application of this new type of biocide, a more comprehensive analysis is needed, such as toxicity evaluation on reproductive and developmental organs, and its potential carcinogenicity in the human body [51, 52]. When applying a WE for air disinfection system, the agents can affect not only the respiratory mucosal membrane but also the skin or superficial mucosal areas, such as the eyes. Thus, a more thorough investigation is required to understand the potential impact of WE on various mucosal surfaces.

In conclusion, our study validates the immunopathological response to inhaled WE, a potent biocidal agent that sterilizes the air around an individual. This validation was achieved by employing analytical methods ranging from transcriptome analysis to clinical symptom assessment in an intranasal C57BL/6 mouse model. These results demonstrate the safety of WE, a recently described potent air disinfectant for human use.

## Conclusions

WE, a novel non-chemical biocide effective against airborne pathogens, requires investigation for its safety and potential inhalation toxicity before practical application. To address these concerns, we evaluated the immunopathological response to WE through an intranasal challenge C57BL/6 mouse model, comparing it with SH, a recognized toxic biocidal agent under inhalation conditions.

Our findings demonstrate that WE did not induce pathological immune reactions in mice. No changes in body weight or production of inflammatory cytokines were detected. Histopathological analysis revealed no lung tissue damage, in contrast to the effects observed in mice treated with SH. Transcriptomic analysis further confirmed the absence of a pathological immune response.

The intranasal challenge of the C57BL/6 mouse model provided valuable insights into the impact of WE at an in vivo level. Moreover, WE appears to be a safe method for eliminating airborne pathogens, with minimal impact on immune system activation and pathology in the C57BL/6 mice.

### Supplementary Information

Supplementary Material 1

## Data Availability

The datasets used and/or analyzed during the current study are available from the corresponding author upon reasonable request.

## Abbreviations

ACK: Ammonium–Chloride–Potassium
ALI: Acute lung injury
ARDS: Acute respiratory distress syndrome
BAL: Bronchoalveolar lavage
CFU: Colony forming unit
CD: Cluster of differentiation
CD11b: Cluster of differentiation molecule 11b (Integrin alpha M)
CD11c: Cluster of differentiation molecule 11c (Integrin alpha X)
COVID-19: Coronavirus disease 2019
DEG: Differentially expressed genes
DW: Distilled water
FACS: Fluorescence-activated cell sorting
GO: Gene ontology
H&E: Hematoxylin and eosin
IL-1β: Interleukin-1β
IL-6: Interleukin-6
IL-12p40: Interleukin-12 subunit beta
LB: Luria Bertani
Ly6C: Lymphocyte antigen 6 complex
Ly6G: Lymphocyte antigen 6 family member G
MERS-CoV: Middle East respiratory syndrome-related coronavirus
SARS-CoV: Severe acute respiratory syndrome coronavirus 2
SH: Sodium hypochlorite
Siglec-F: Sialic acid-binding Ig-like lectin-F
TNF-α: Tumor necrosis factor-alpha
WE: Water electrospray
