# Supplementary material for: Assessment of immunopathological responses of a novel non-chemical biocide in C57BL/6 for safe disinfection usage
Source: Lab Anim Res. 2024 Aug 13;40:28. doi: 10.1186/s42826-024-00214-6 (PMC11320990; doi:10.1186/s42826-024-00214-6)
Supplement: Supplementary file 1 — Supplementary Material 1 [file 42826_2024_214_MOESM1_ESM.pdf]

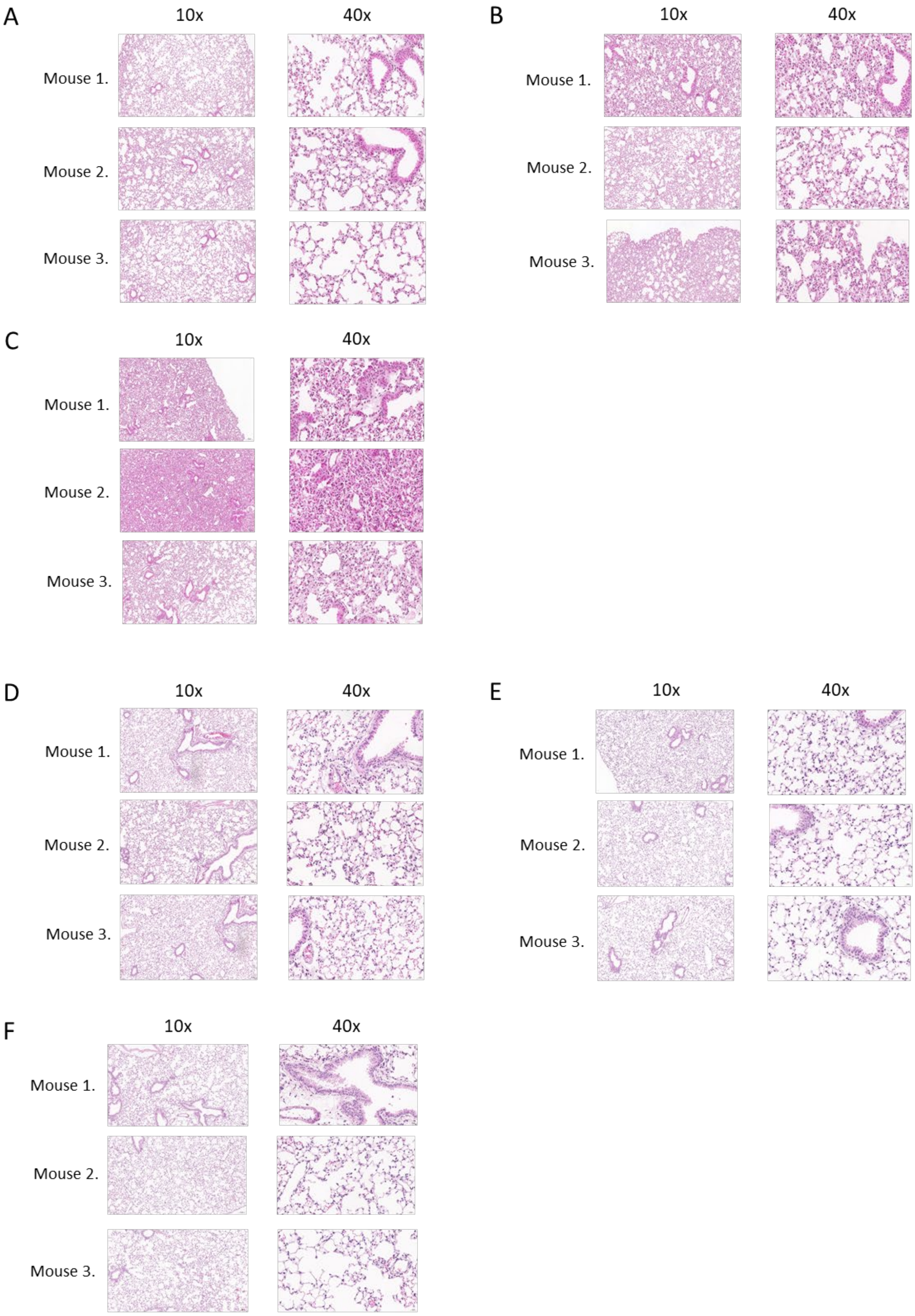

Supplementary Fig. 1. Histopathological analysis of WE and other bactericidal agents. Histopathological analysis was conducted using H&E staining on samples from mice treated with distilled water (DW, n=3), water electrospray (WE, n=3), and sodium hypochlorite (SH,

n=3).

(A–C) Three representative images from each mouse at day 10, including (A) DW–, (B) WE–, and (C) SH–treated mice.

(D–F) Three representative images from each mouse at day 21, including (D) DW–, (E) WE–, and (F) SH–treated mice.

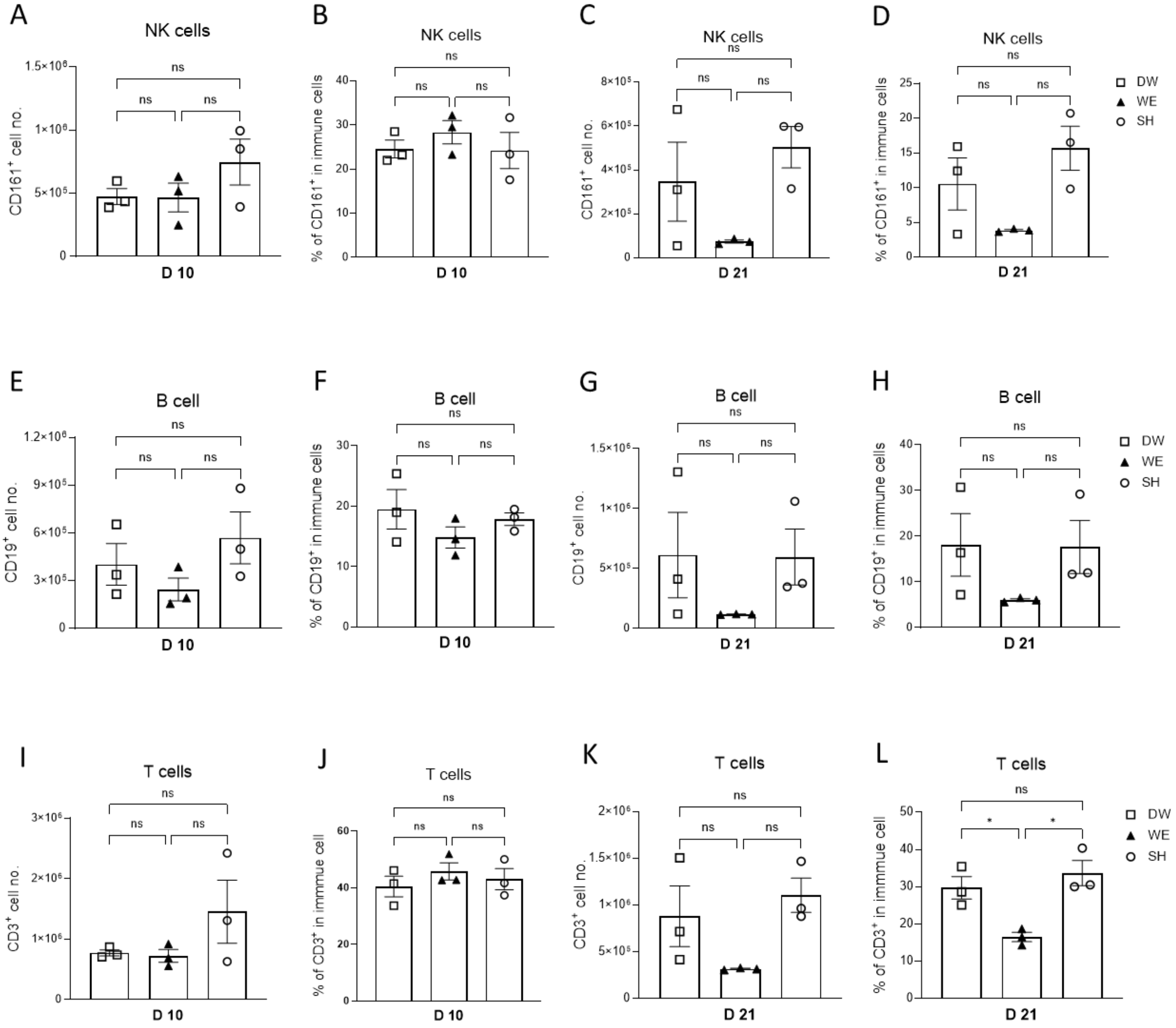

Supplementary Fig. 2. Lung tissue–infiltrating lymphoid immune cell analysis of WE–treated mice by flow cytometry.

The lung resident and infiltrating immune cells of mice treated with distilled water (DW, n=3), water electrospray (WE, n=3), and sodium hypochlorite (SH, n=3) were analyzed using flow cytometry.

(A–D) The frequency and total number of $CD161^+$ NK cells were quantified in lung samples at (A, B) day 10 and (C, D) day 20.

(E–H) The frequency and total number of $CD19^+$ B cells were quantified at (E, F) day 10 and (G, H) day 20.

(I–L) The frequency and total number of $CD3^+$ T cells were quantified at (I, J) day 10 and (K, L) day 20.

The bar plots show the mean ± SEM; the dots represent individual mice. *p* values are from one-way ANOVA. * $p < 0.05$, ** $p < 0.01$, *** $p < 0.001$.

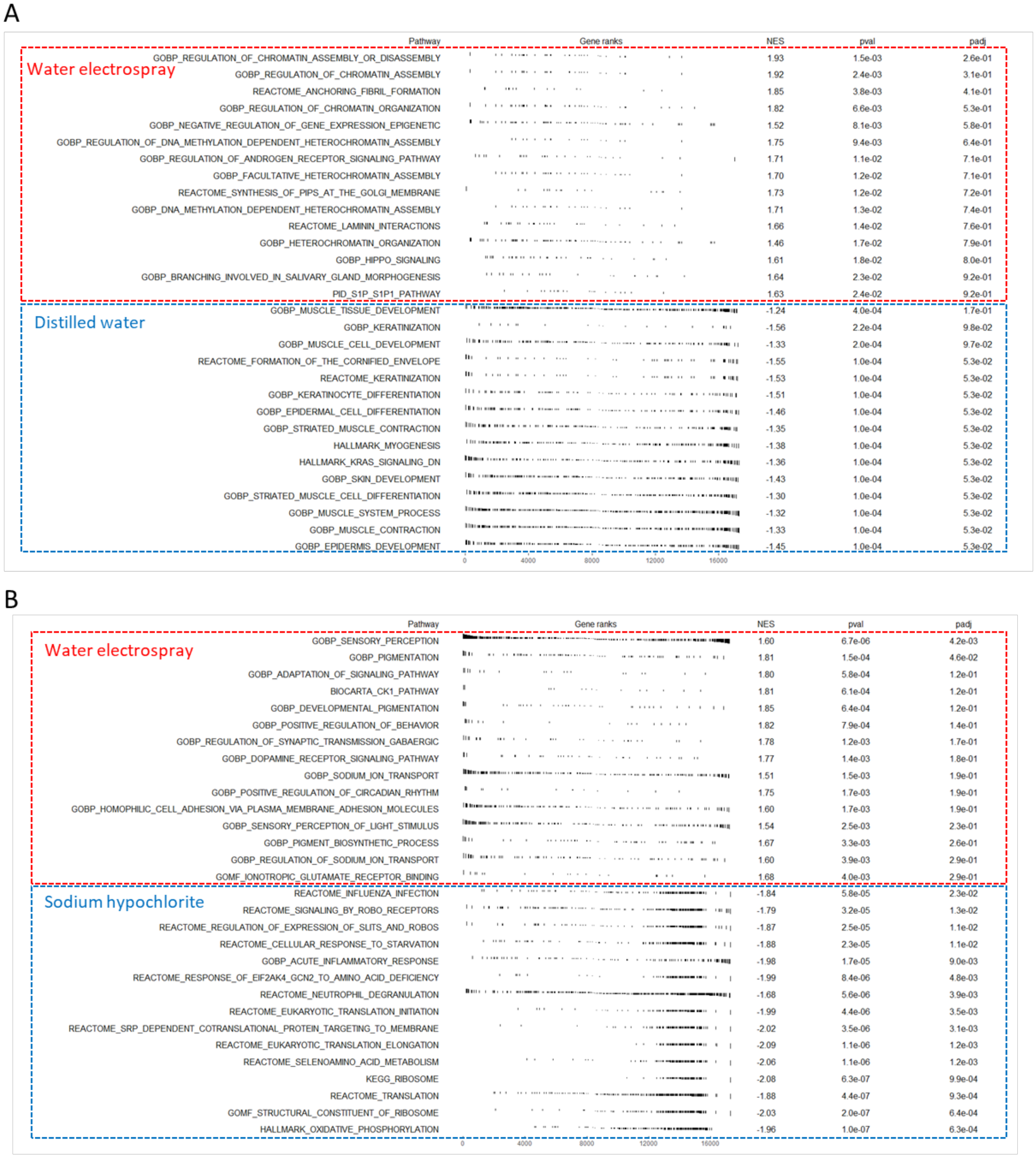

Supplementary Fig. 3. Gene set enrichment analysis of mice treated with WE versus (A) DW and (B) SH.

Table 1. Histopathology scoring system

Alveolar involvement	Histopathological score
None	0
Mild (Focal increase cellularity without septal thickening)	1
Moderate (Increased cellularity with septal thickening)	2
Severe (25~50% visualized lung with increased cellularity & thickening)	3
Diffuse (>50% visualized lung with increased cellularity & thickening)	4
